# A Stretchable Electronic Tattoo for Self-Powered Human–Machine Interfaces and Therapeutic Applications

**DOI:** 10.3390/mi17030312

**Published:** 2026-02-28

**Authors:** Rumeng Shao, Yixuan Zhang, Ya Chang, Chuanbo Li, Yang Wang

**Affiliations:** 1 School of Science, Minzu University of China, Beijing 100081, China; 2 Optoelectronics Research Centre, Minzu University of China, Beijing 100081, China

**Keywords:** electronic skin, self-powered device, smart tattoos, wearable electronics, graphene

## Abstract

Flexible skin electronics are increasingly sought after for their potential in sensing and drug delivery within wearable human–machine interfaces. However, developing multifunctional applications that maintain biocompatibility and stable electrical performance under various mechanical deformations remains a challenge. Here, we introduce tattoo paper-based graphene–gold conductors that are approximately 0.04 mm thick and feature a dual conductive pathway within the graphene–gold film. By integrating a folding structure with this dual conductive pathway, we can mitigate the strain effects on the electrical resistance of film-based conductors, resulting in wider areas of stable resistance. In addition, we have designed film conductors with a kirigami structure, which achieves a high initial conductivity of 1.5 × 10^3^ S cm^−1^ and exhibits negligible resistance changes across a broad strain range of 0 to 130%. We utilize these conductors to develop waterproof on-skin patches that incorporate electrically and optically active heaters for body heating and drug delivery. Furthermore, we have created an on-skin dialing interface using these conductors, which enables users to make telephone calls based on triboelectric nanogenerators.

## 1. Introduction

Tattoo-like electronics that are thin and highly conformable hold significant promise for applications in clinical diagnostics and human–machine interfaces [1,2,3,4,5,6,7]. These devices should maintain stable conductive properties even after deformation and create a seamless contact with the skin or irregular surfaces. Typically, epidermal electronics are manufactured using flexible polymers, such as polydimethylsiloxane (PDMS) [8,9], polyimide [10], polyurethane [11], or fluorine rubber [12,13], along with conductive fillers like nanowires [14,15], carbon nanotubes [16,17], or graphene. These materials are processed using conventional methods such as high-temperature techniques, chemical vapor deposition, and electrospinning. Despite some exciting developments, the trade-off between multifunctional applications and low preparation costs may impede the progress of tattoo-like electronics.

Utilizing thin metal films on polymeric substrates can help achieve high conductivity while the electronics are stretched or twisted, addressing some of the above issues. Existing studies to improve the resilience of metal electrodes fall into two strategies. One focuses on a structural design, such as tines, wrinkles, and network cracks [18,19,20,21,22,23,24,25]. The other method relies on intrinsically stretchable materials, including metal nanowires and liquid metals [26,27,28,29,30,31,32,33,34,35,36]. For example, Cho et al. demonstrated a flexible metal conductor with a strain-resilient performance, which can be improved using a two-dimensional interlayer [37]. However, most metal films have low optical absorption, which reduces their multifunctional applications for photothermal therapy. Therefore, it remains a challenge to develop a readily available method to obtain highly conductive, low-reflective, strain-resilient, and adhesive thin-film electrodes.

Here, we proposed a cost-effective approach to accomplish the mechanically strain-resilient electrical performance of metal film-based conductors, which are made from graphene–copper composite film on folding tattoo paper. By using the kirigami technique, we pioneered flexible thin electrodes that exhibit a high strain-insensitive conductivity (~1.5 × 10^3^ S cm^−1^) over a tensile strain range from 0% to 130%. Due to their thin thicknesses and adhesion properties of tattoo paper, the electrodes can form contact with different material substrates, including polydimethylsiloxane (PDMS), glass, metal, fiber, and human skin. Following this approach, we constructed an on-skin intelligent communication system for calling a cellphone and created a thermal patch for heating skin by electrical and optical signals. We hope the proposed approach has a broad future in flexible electronic devices.

## 2. Materials and Methods

### 2.1. Materials

Graphene water-based paste was purchased from Tanfeng Graphene Technology Co., Ltd. (Suzhou, China). copper conductive paint (DD001) was supplied by Jingzhe Intelligent Technology Co., Ltd. (Shenzhen, China). tattoo paper was purchased from Maggie Technology Co., Ltd. (Shenzhen, China). PDMS (SYLGARD 184) and PET films were obtained from Dow Corning (Shenzhen, China) and Dupont company (Zhejiang, China), respectively. Silver pastes and copper wires were purchased from Yingxun Technology Co., Ltd. (Guangzhou, China).

### 2.2. Fabrication of Layered Cu–Graphene Electrodes

Firstly, we prepared graphene solution by dissolving graphene paste (1 mL) in 4 mL of deionized water and stirring for 20 min at room temperature. Then, the graphene solution was blade-coated on tattoo paper at 10 mm/s. The copper paint was sprayed on the prepared graphene film. Finally, the tattoo film was coated on the Cu–graphene film. The tattoo film can easily adhere to various substrates, including PET film, skin, and PDMS film. The PDMS film was fabricated by mixing the base and crosslinking agent at a weight ratio of 10:1 and removing bubbles by vacuuming. To adhere the layered Cu–graphene film on these substrates, we soaked the tattoo paper in the deionized water and peeled it off from the substrate. The kirigami structures were fabricated via the laser engraver (M1, xTool, Shenzhen, China).

### 2.3. Electronic Skins

Electronic tattoo paper was cut into the designed array and attached to hands for the on-skin devices. To build the electrical contacts, we bonded fine copper wires to the Cu–graphene surface of the electronic tattoo paper using Ag paste.

### 2.4. FEA Simulation

The stress and deformation field distribution of the films and potential distribution of the self-powered E-skin were established using the finite element method in commercial software (COMSOL Multiphysics 5.0). The values of the geometric parameters were determined based on the fabricated devices and the relevant material parameters were obtained from the COMSOL built-in database. The fracture behavior was simulated using a solid mechanics module, with copper thin-film material parameters selected from the standard library. Based on experimental observations, two distinct crack geometries were modeled: (1) a through-thickness crack with a defined length of 400 μm; (2) an irregular fracture pattern with a defined length of 100 μm. A tensile load of 50 kPa was applied at both boundaries to evaluate the mechanical response.

### 2.5. Characterizations

The morphologies of the Cu and graphene film were characterized with a scanning electron microscope (HITACHI, S-4800, Tokyo, Japan) and optical microscope (LEICA, 2700P, Wetzlar, Germany). Raman spectroscopy was performed by Evolution with 532 nm laser excitation (Horiba, HR-800, Kyoto, Japan). The output electrical signals of the electrode were collected using a digital meter (Keithley, 2400, Cleveland, OH, USA), and the voltage change was measured by a Keithley DAQ6510 multi-meter system (Cleveland, OH, USA). The thermal heating of the heaters was collected by a thermal-imaging camera (FOTRIC, 240, Shanghai, China).

## 3. Results and Discussion

### 3.1. Design Principle and Mechanism

**Figure 1:**
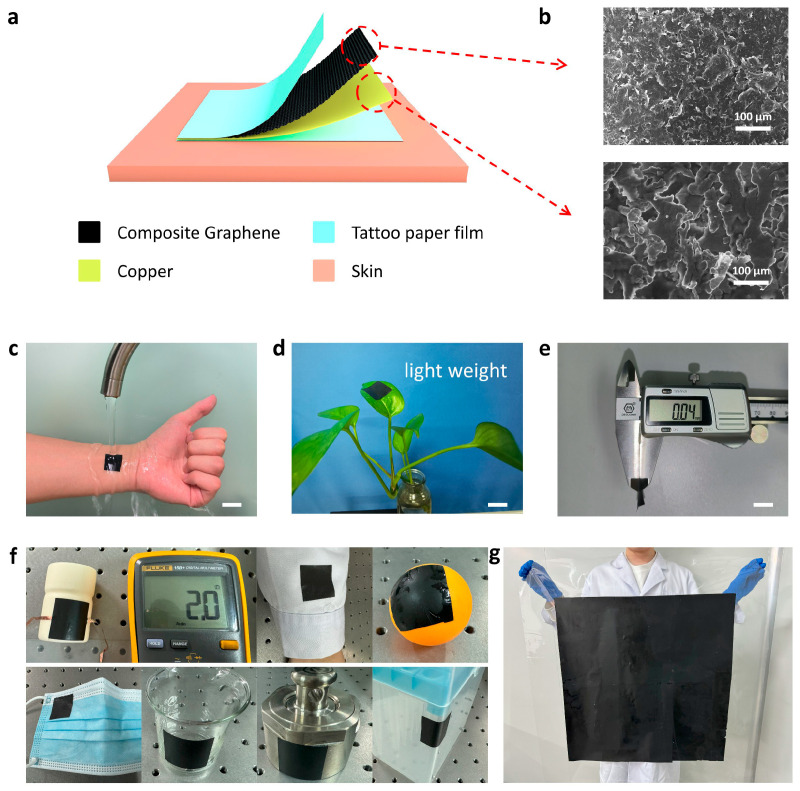
Design of flexible layered conductors. (**a**) Structural illustration of the Cu–graphene film-based conductor. (**b**) SEM image of graphene and Cu films. (**c**) Illustration of the adhesion measurement between the electrodes and skin. (**d**) Photograph of a flexible conductor with a dimension of ~2 cm × 2 cm, standing on a leaf, indicating its light weight (scale bar: ~2 cm). (**e**) Thickness of the conductor, indicating its thinness. (**f**) Illustration of extensive adhesion to a variety of substrates. (**g**) Photograph of large-area conductive film.

Figure 1a presents the schematic diagram of the flexible on-skin electrodes with a layered architectural design. We adopted commercial conductive materials, including graphene ink and copper paint, to fabricate the conductive layer, which is low-cost and machinable. The fabrication process is detailed in the methods (Figure S1). The graphene film was prepared on the tattoo paper using a simple blade-coating method. The copper paint was sprayed on the graphene film and was covered by the adhesive film. Then, the on-skin electrode was completed by tattooing it onto the substrate and peeling the top piece off the substrate. The scanning electron microscopy (SEM) images indicate that the graphene materials comprise a mass of nanosheets; the metal film surface adopts a random convex morphology induced by the crosslinking between copper nanoparticles and dispersed polymers during the spraying process (Figure 1b). Cross-sectional SEM images show ultrathin layers of the graphene–copper with a thickness of about 3.5 µm (Figure S2). The prominent features that appeared in the Raman spectra of the ink are connected to graphene (Figure S3).

The tattoo paper made it easy to form a seamless contact between the on-skin electrodes and substrates that were resistant to water rinsing (Figure 1c). The on-skin electrode is very light (~5 mg) and thin (~0.04 mm), making it easy to support by greenery without any collapse (Figure 1d,e). These thin conductors can be fabricated on various substrates, such as glass, fiber, ceramic, and plastic, because of the excellent adhesive and deformable features of tattoo papers (Figure 1f). Moreover, our cost-effective approach can achieve the large-area preparation of flexible electrodes, indicating its wide range of practical values (Figure 1g). Adhesion tests were performed on various substrate surfaces, revealing a significant influence of the substrate materials on the adhesive properties of the thin-film electrodes. The electrodes demonstrated favorable adhesion to skin surfaces. However, reduced adhesion strength was observed on flexible substrates, primarily attributable to their characteristically smooth surfaces and low surface energy (Figure S4).

### 3.2. The Electromechanical Performance of the Flexible Electrodes

To explain how the Cu–graphene composite films can be utilized, we investigated various conductive materials, such as a carbon nanotube, graphene, copper particles, pencil, and poly (3,4-ethylenedioxythiophene) (PEDOT). These materials can be fabricated on tattoo papers by using the scraping method. Compared with other conductive films, the electrical resistance of the graphene film increased gradually upon bending, indicating excellent strain insensitivity (Figure S5). However, the graphene film presented high resistance because the graphene solution usually contained surfactant, which could affect the resistance of the film (Figure S6). To achieve low resistance and high mechanical stability, we selected the graphene film as the interlayer between the low-resistance Cu film and the tattoo paper to restrain cracks on the Cu film and establish a double conductive path (Figure S7). Under uniaxial tensile loading, the thin-film electrode exhibited fracture initiation at 3% strain, with subsequent propagation leading to complete mechanical failure (Figure S8).

Another critical parameter for achieving resistance bending strain insensitivity is the small folding structures of tattoo papers, which reduce partial adhesion between the tattoo paper and flexible substrate (Figure S9). We bent the tattoo paper to engineer folding structures before transferring the modified tattoo paper to the substrates. Compared with bare conductive film, conductive film established on tattoo paper is more electrically stable because folding structures could restrain transversal cracks on the conductive films due to adjusting the internal stress during the bending process.

**Figure 2:**
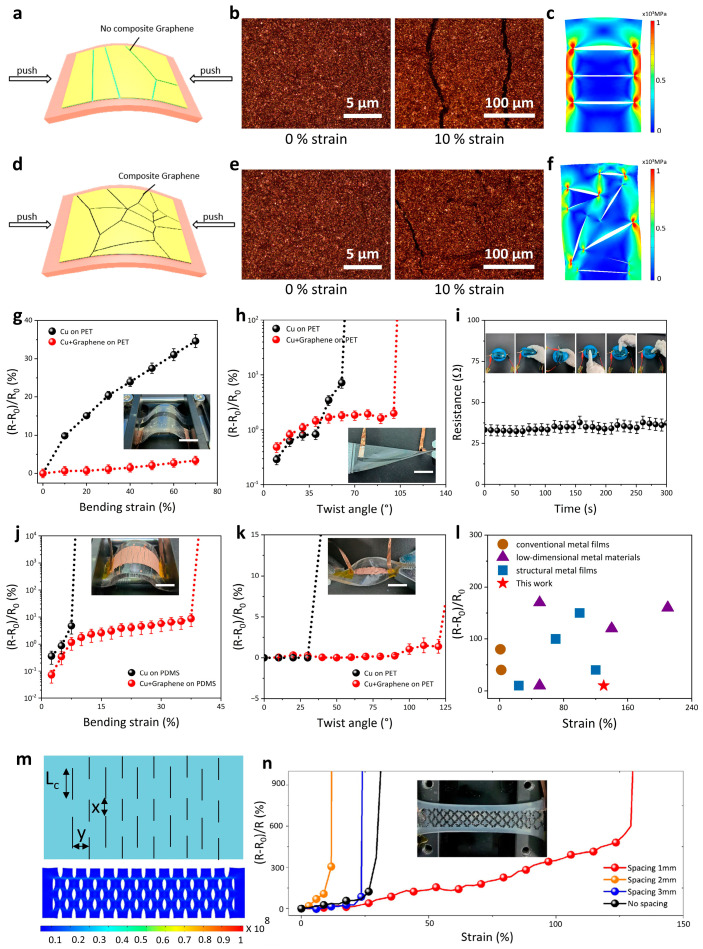
Mechanism of microcracked morphology structure and electrical characterization. (**a**) Schematics of crack mods on the bare Cu film. (**b**) Optical microscope images of surface morphologies of the Cu film at tensile strain. (**c**) Simulated stress distributions of the Cu film. (**d**) Schematics of crack mods on the bare Cu–graphene film. (**e**) Optical microscope images of surface morphologies of the Cu–graphene film at tensile strain. (**f**) Simulated stress distributions of the Cu–graphene film. (**g**) Electrical behaviors of Cu and Cu–graphene films on PET film (1 mm) in response to bending deformation. (**h**) Electrical behaviors of Cu and Cu–graphene films on PET film in response to twist deformation. (**i**) Resistance response of Cu–graphene films on balloon under different mechanical deformation conditions. (**j**) Electrical behaviors of Cu and Cu–graphene films on PDMS film (4 mm) in response to bending deformation. (**k**) Electrical behaviors of Cu and Cu–graphene films on PDMS film in response to twist deformation. (**l**) Comparison of relative resistance change at different strains with other metal-based flexible conductors. (**m**) Theoretical mechanical models and simulated stress distribution for the kirigami pattern. (**n**) Resistance changes in the conductive films with different kirigami patterns under different stretching conditions.

Fundamentally, we combined a flexible tattoo paper with folding structures with graphene film adopted to adjust the in-plane fracture modes, which can restrain the crack propagation of the copper film. We fabricated two types of on-skin electrodes and characterized the in-plane fracture behavior, illustrating the mechanism for enhancing strain tolerance (Figure 2a). Straight cracks occur with a bare Cu film on tattoo paper without folding structures, as shown in Figure 2b. This primary cause is the mechanical disparity between metal films and soft substrates. Figure 2c shows the stress distribution of the electrode surface under stretching. The stress mainly appears on the edge of the cracks, which could cause an integral fracture of the conductive film. In the presence of Cu–graphene layered films on the tattoo paper with folding structures, the in-plane crack development of the copper film under bending is modified, resulting in a significant degree of irregular deflected cracks (Figure 2d,e). These tiny cracks can reduce the tensile strain, improving the mechanical robustness of the film (Figure 2f). These results show that the yield strain of the flexible film can control the crack formation of the metal film [32,38]. In addition, the layered Cu–graphene composite design can achieve two channels for electron transport, which could eliminate strain effects on the conductive performance of the electrodes. We propose that this bilayer structure can prevent the formation of cracks through two mechanisms. Firstly, the micro-nano structures on the copper surface create an interconnected architecture with graphene. Secondly, the wrinkled deformation of graphene helps to release the strain energy. The graphene coating functions as a conductive bridge, establishing alternative current pathways when microcracks develop in the copper film. We investigated the surface morphology and electrical conductivity of various electrodes. A primary limitation of our approach is the insufficient controllability over surface cracking, which leads to variations in crack patterns. Nevertheless, this method effectively generates random microcracks, suppresses the formation of through-thickness cracks, and utilizes the graphene layer to bridge cracks within the same plane. As a result, it ensures stable conductivity under mechanical stretching for electrodes fabricated on diverse substrates.

To investigate the strain insensitivity of the flexible electrodes, we characterized the resistance as a function of the bending strain, with the tattoo paper used as an adhesive layer for different substrates. The resistance of the Cu–graphene electrodes increased slowly on the polyethylene terephthalate (PET) film upon bending, in contrast to the abrupt, apparent increase in resistance observed in the bare Cu electrodes (Figure 2g). Meanwhile, the electrodes showed enhanced strain-resilient electrical to twisting angles (Figure 2h). Next, we tattooed the flexible electrodes onto the surface of a balloon and tested its resistance to possibly static pressures. Figure 2i shows that there is almost no change in the resistance. These results indicated the flexible Cu–graphene electrode not only exhibits stable electrical conductivity to different strains but is also suitable for various substrates.

Considering the concern of thick flexible substrates, the strain-resilient electrical performance of the flexible on-skin electrodes was investigated on the PDMS substrate. Figure 2j shows the electromechanical responses of the Cu and Cu–graphene electrodes on PDMS. In this experiment, the electrical failure of the bare Cu electrode arises at ~7% tensile strain as a sharp conversion due to the quick formation of linear cracks. For the Cu–graphene electrodes, the relative resistance changes remain stable within the strain range from 0% to 37.5%, which is attributed to the formation of irregular deflected cracks and two channels of copper and graphene for electron transport. The conductive film exhibits minimal hysteresis at low tensile strains (<24%), with negligible resistance variation during both the stretching and recovery phases. However, as the strain increases beyond this threshold, the proliferation and expansion of microcracks lead to rapid resistance escalation. During strain recovery, interfacial mismatch between these cracks and the flexible substrate material results in significant hysteresis (Figure S10). After being twisted to an angle of about 120°, the Cu–graphene electrode showed stable electrical conductivity (Figure 2k). It exhibited good mechanical durability under over 10,000 loading cycles at 16% bending strain (Figure S11). Additionally, the electrodes can form seamless contacts with human skin and show advantageous electromechanical robustness, where a light-emitting diode (LED) maintains stable brightness during finger bending (Figure S12).

To further extend the stretchability of the Cu–graphene electrodes, we fabricated the uniaxial structure by using the kirigami method, a paper-cutting art [39,40]. As illustrated in Figure 2m, the uniaxial pattern consists of parallel lines in a configuration of rectangles. The pattern involves three essential parameters: x is the interval of cut ends, y is the vertical distance, which can restrain the stretchability of the conductive film, and L*c* is the cut length, which improves the stretchability. Figure 2m shows the stress distribution of the pattern under stretching, indicating no apparent concentration. During the deformation process, the shear pattern undergoes three primary deformation stages. In the first stage, the structure composed of parallel and symmetrical incisions experiences external loading, causing each beam to rotate around its nodal points and bend in-plane, thereby elongating the entire shear sheet along the stretching direction. In the second stage, the beams continue to deform along the tensile direction, with stress rapidly increasing until fracture initiation occurs. The third stage is characterized by beam fracture, where cracks progressively accumulate, ultimately leading to complete structural failure. Through experimental investigation and theoretical analysis of three distinct shear patterns, we conclude that an increase in the *y* of the shear configuration results in a more compact tensile structure, thereby reducing its stretchability. Conversely, an increase in the L*c* leads to a looser tensile structure, enhancing its stretchability (Figure S13). As a result, the relative resistance changes in the Cu–graphene electrodes with the uniaxial pattern remain almost constant within the tensile strain range from 0% to 130% (Figure 2n).

The kirigami-patterned conductive films exhibit significantly reduced hysteresis compared to their conventional thin-film counterparts (Figure S14). The electrical stability of five fabricated thin films was examined under tensile strain. The results showed consistent electrical stability and reproducibility across all samples, highlighting the high repeatability of the fabrication method. Additionally, future studies will utilize an automatic coating machine for large-scale production to meet the demands of practical applications in larger areas (Figure S15). We compared the mechanical performances of our Cu–graphene electrodes with advanced metal-based flexible conductors, presenting outstanding performance within the same strain range (Figure 2l) [37,41,42,43,44,45,46,47,48,49]. These characteristics are crucial for their practical utilization in wearable electronic devices. Compared to these studies, our fabrication method is more straightforward and rapid, enabling the stable construction of electrodes on various substrate surfaces. The resulting electrodes are lightweight, thin, and exhibit excellent conformability. These electrodes can serve as conductive layers in flexible electronic devices—such as flexible photothermal devices and tactile sensors—meeting the demands for comfortable wearability. Currently, the as-fabricated conductive films show stress concentration at the nodal regions when subjected to biaxial tension. This stress could cause simultaneous failure of both the copper film and the graphene, potentially resulting in cracks that propagate through the thickness of the material. To address this issue, we aim to improve the electrical stability of the conductive films during biaxial stretching by optimizing the kirigami architecture, including serpentine cuts and spring interconnects.

### 3.3. Self-Powered Sensors Enabled by On-Skin Electrodes

**Figure 3:**
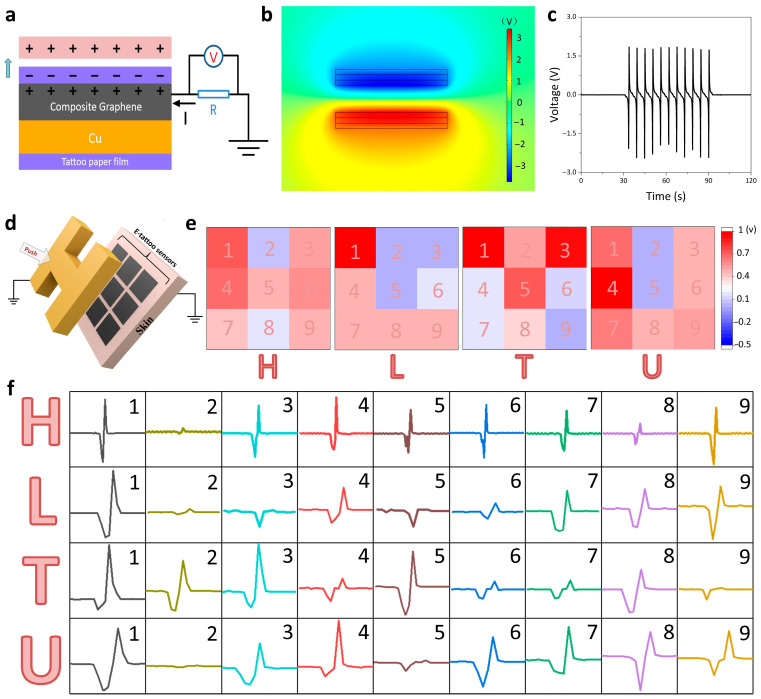
Mechanism and electrical signals of the Cu–graphene film based on self-powered E-skin. (**a**) Schematic illustration of the working principle for the Cu–graphene film as an E-skin based on the TENG principle. (**b**) Simulated potential distributions of the Cu–graphene film as an E-skin. (**c**) Output voltage of the Cu–graphene film (3 cm × 3 cm) under the impact of a fluorinated ethylene propylene film. (**d**) Schematic of obtaining tactile information upon touching the E-skin sensor array with the subject. (**e**) The 3 × 3 voltage pixels of E-skin sensor elements under the impact of different letters. (**f**) Corresponding voltage signals for each element under the impact of letters (“H,” “L,” “T,” and “U”).

The design of on-skin electrodes enables them to function as a self-powered device based on the principle of the triboelectric nanogenerator (TENG). The TENG has been explored as a self-powered sensor for human–machine interaction and health care monitoring [50,51,52]. Figure 3a schematically illustrates the working mechanism of the triboelectrification effect originating from the displacement current. The tattoo paper film and a PDMS film serve as the positive friction material and the negative friction material, respectively. When the PDMS film and tattoo paper film come into contact with each other, electrons flow from the tattoo paper film to the surface of PDMS owing to the difference in their electronegativities. Once they separate from each other, electrons would flow from the ground to on-skin electrodes to equilibrize the contact potential difference between the two layers, resulting in a positive output signal. As the PDMS further detached from the on-skin electrode, the negative charges on the surface of the PDMS film were fully screened from the induced positive charges on the surface of the tattoo paper film, leading to no generation of an output signal. When the PDMS approached the on-skin electrode again, electrons would transfer from the electrode to the ground, producing a negative output signal. Therefore, this continuous process of contact and separation could generate a sequence of alternating output signals. Using a finite element analysis (FEA), we can illustrate the potential difference between the two films (Figure 3b). Figure 3c shows the obtained voltage under a load of 100 MΩ with a contact area of 2 cm × 2 cm. Compared with traditional pressure sensors, including piezoresistive and capacitive sensors, our ultrathin sensors can detect pressure stimuli without an external power supply. The device generates AC pulse signals with low response and recovery times during the contact separation process with external pressure (Figure S16). These advantages make it a promising option to fabricate wearable electronics.

To utilize the above function as a sensing platform, we fabricated the 3 × 3 sensor array, where each element can generate output values due to the touch stimuli (Figure 3d). When a subject is in contact with the surface of the array, the touch cognition can be mapped by estimating the output voltages of each domain. During human motion, the device generates low-amplitude voltage fluctuations (10–50 mV). In contrast, physical contact with external objects produces significantly enhanced triboelectric output signals (1–2 V). This substantial magnitude difference facilitates reliable signal discrimination through subsequent hardware circuit processing (Figure S17). We plotted the data using absolute values; the depth of red represents the peak magnitude, and the red regions are aligned with the corresponding letters. Figure 3e illustrates the voltage mapping based on the foam of different shapes, including “H,” “L,” “T,” and “U.” The actual signals of the sensor array correspond to the letters, indicating that the array can detect the applied pressure of each element (Figure 3f). Hence, our layered design of on-skin electrodes is a promising candidate for human–machine interfaces.

### 3.4. Thermal Stimulations of On-Skin Electrodes

**Figure 4:**
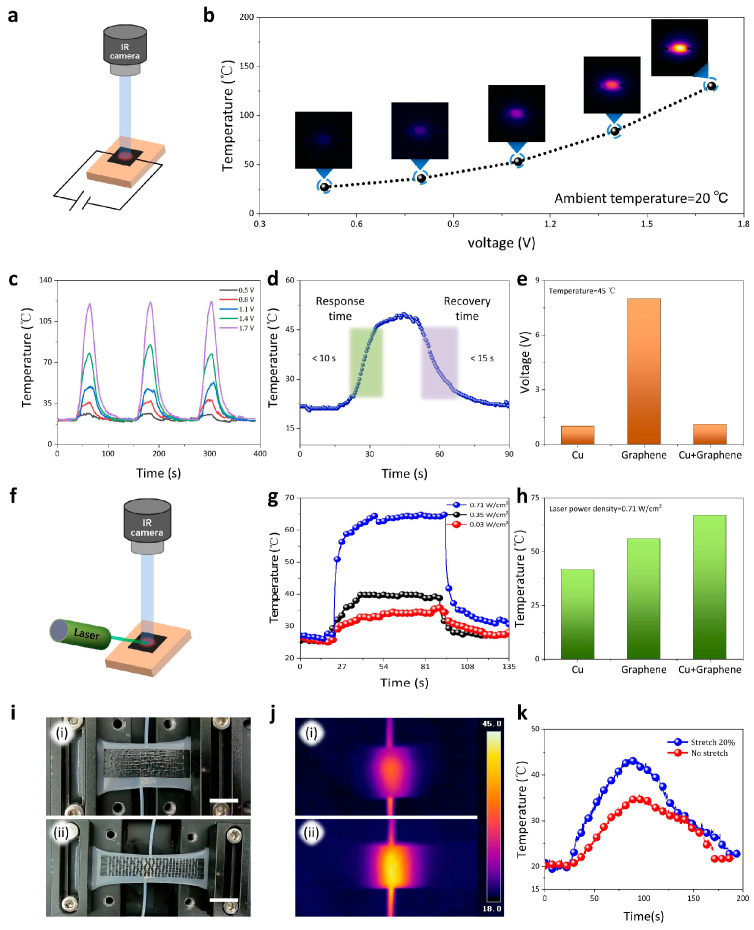
Electrical and optical heating performance of the Cu–graphene film as a heater. (**a**) Schematic illustration of an electrically induced heating element. (**b**) Heater response as a function of incident driving voltage. (**c**) Temperature–time profiles of the joule heating heater with different applied voltages. (**d**) The response and recovery time of the heater under the ON/OFF switch in the circuit. (**e**) Driving voltage with different conductive films at 45 °C. (**f**) Schematic illustration of an optically induced heating element with 532 nm laser illumination. (**g**) Transient heater temperature at incident optical power densities. (**h**) Heater temperature of different conductive films with 0.71 W/cm^2^. (**i**) Optical image of heat dissipation for the conductive films with kirigami patterns. (**j**) IR images of the films under different stretching conditions, with (**i**) and (**ii**) corresponding to 0% and 20% strain, respectively. (**k**) Transient temperature profile when the film is under different stretching.

We systematically investigated the joule heating effect of on-skin electrodes, a potential application in thermotherapy (Figure 4a). Tattoo paper does not generate thermal energy due to its electrical insulation. When it is electrically stimulated, the Cu–graphene film can maintain equal thermal distribution, as shown in the infrared (IR) thermal image (Figure S18). Joule heating is driven by electrical power ($P_{Joule}$), calculated as PJoule=V2R, where *V* is the applied voltage and *R* is the resistance of the Cu–graphene conductors [53]; the electrical power was, where *R* is the resistance of the Cu–graphene conductors. As the voltage increased, the obtained temperature gradually increased, reaching 120 °C at a low voltage of ~1.7 V (Figure 4b). Furthermore, the on-skin electrode has stable cyclic responses under different applied voltages ranging from 0.5 V to 1.7 V, indicating a wide heating range for our devices (Figure 4c). As the applied voltage gradually increases (0.5 V–1.7 V), the current passing through the thin film exhibits a corresponding rise, demonstrating stable conductivity of the thin-film electrode under varying voltage conditions (Figure S19).

Figure 4d shows the temperature–time profiles with three regimes, including temperature rising, thermal equilibrium, and temperature falling. The rise and decay times were 10 s and 15 s, indicating that our device can fast heat heating subjects and recover the room temperature. We also investigated the heating performance for different electrodes and found that the Cu–graphene and Cu electrodes need a lower voltage than the graphene electrode to achieve 45 °C (Figure 4e). These results indicate that our on-skin electrodes, to control the heating temperature, required a lower driving voltage than other skin heaters [54].

Heating via the external power source may be subject to the application environment, which makes it hard to establish the electrical contact for under-tissue heating. Utilizing the photothermal effect can also increase the surface temperature of our on-skin electrodes (Figure 4f). We used a 532 nm green laser to illuminate the electrode and measured the heating temperature. Figure 4g shows that the stabilized temperature can be enhanced by increasing the power density, where it can achieve ~65 °C at the power density of 0.71 W cm^−2^. Compared with the Cu and graphene electrodes [14,17], the Cu–graphene electrodes displayed superior heating efficiency, which should result from the strong optical absorption and high conductivity of the layered electrodes (Figure 4h). We assessed the long-term wear stability of the device through a 12 h test under real-life conditions, which included exposure to water and perspiration. The results showed no noticeable signs of skin irritation, allergic reactions, or thermal erythema at the contact sites (Figure S20). These results make our on-skin electrode applicable to on-point thermotherapy in medical applications [55].

Next, we investigated the heat-managing performance of the Cu–graphene electrodes with the uniaxial structure on flexible silicone substrates. The electrode was fixed on a small stretching table, and a heating plate was put under it (Figure 4i). The representative thermal infrared camera pictures obtained from the electrode before and after stretching are shown in Figure 4j. The images revealed that flexible electrodes under tensile strain had a better heat dissipation. In this state, the temperature of the object would reduce quickly because the tensile seams without Cu–graphene materials would promote radiation. The thin-film electrode was placed on the heating stage. Initially, the electrode demonstrated effective thermal insulation properties. Upon application of tensile strain, heat transfer occurred through the resulting discontinuous micro-gaps, leading to a corresponding increase in the surface temperature. More generally, the temperature observed for the electrode was increased by 6 °C with an increase in 20% tensile strain (Figure 4k). Hence, we can adjust the rate of cooling for the object by changing the tensile strain of the flexible electrode. The tattoo paper possesses advantages of easy cutting and transferability, which facilitate the creation of large-area flexible conductive electrodes on various substrates through a splicing technique. Initially, we constructed graphene–copper bilayer conductive structures on multiple small-area tattoo papers following the proposed fabrication protocol. Subsequently, these tattoo papers were transferred and assembled onto large-area plastic films. The resulting flexible conductive film exhibits remarkable thinness and lightweight properties while effectively blocking human body thermal radiation. Thermal imaging shows that body areas covered by conductive films have noticeably lower temperatures compared to the unshielded regions of the head (Figure S21). We hope this innovative film can find potential applications in military fields, particularly for assisting soldiers in evading infrared imaging surveillance during nighttime operations.

### 3.5. Wearable Applications for On-Skin Electrodes

**Figure 5:**
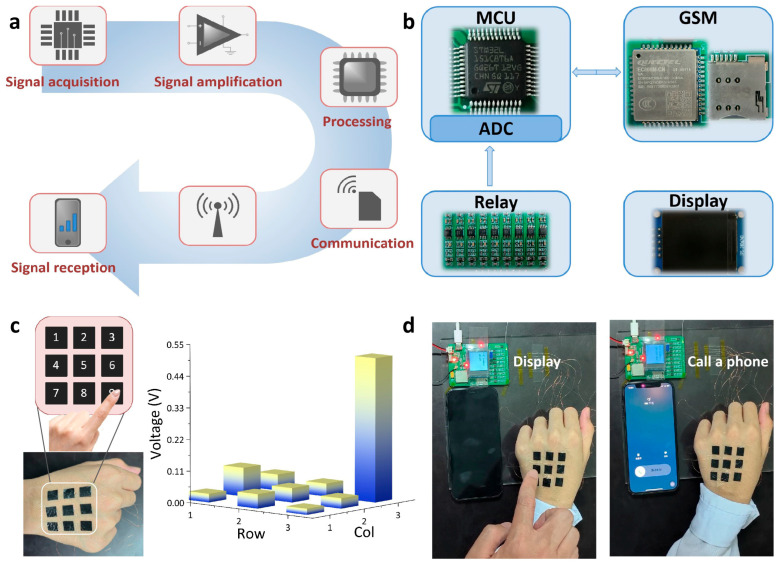
Self-powered E-skin for intelligent electronic device. (**a**) Schematic diagram of the manipulation of a dialing communication system. (**b**) Circuits for signal machining and transmission. (**c**) Optical image of the 3 × 3 E-skin sensor array on the hand; numbers indicating the respective element. Inset showing a 3D diagram of peak voltage upon touching digit 9 with a finger. (**d**) Demonstration of dialing a cellphone by touching one group of digits for an actual phone number via the E-skin sensor array.

As a conceptual application for smart skin, we constructed an innovative dialing communication system, which mainly consists of layered electrodes, a voltage comparator, a microprogrammed control unit (MCU), and a global system for mobile communications (GSM) (Figure 5a,b). The high conductivity, strain-resilient performance, and triboelectric ability of the layered Cu–graphene electrodes allow them to serve as an electronic skin (E-skin) for applications in wearable electronics. Figure 5c shows the corresponding photographs of our system. To realize the dialing function, we fabricated a 3 × 3 sensor array by tattooing the on-skin electrodes on the hand, representing keys 1–9 in the system. When a finger is pressured on the surface of each element, each digit can produce a corresponding voltage signal. After touching a group of elements for an actual phone number, we can call a mobile phone (Figure 5d). Hence, the on-skin electrode with self-powered tactile sensing capabilities is a promising candidate for human–machine interfaces and intelligent robotics.

**Figure 6:**
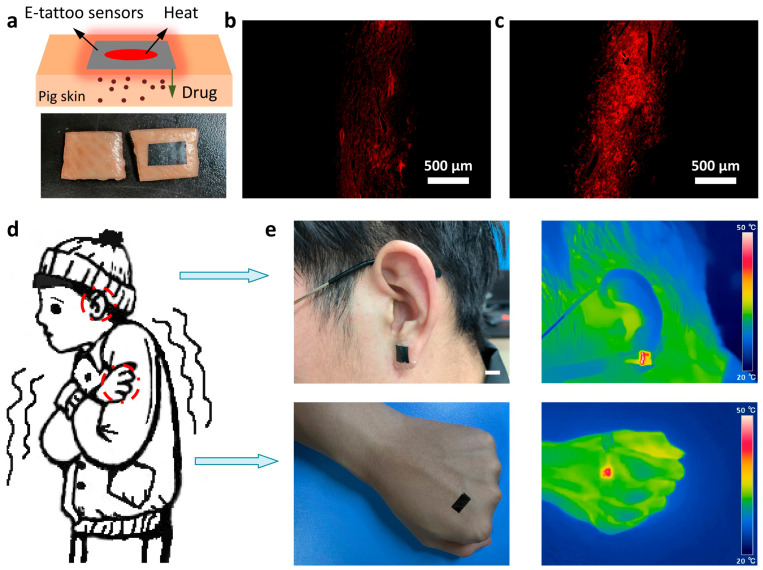
Optical and electrical heating performance of the conductive films. (**a**) Schematic diagram of optically induced heating. The conductive film attached to pig skin. (**b**,**c**) Cross-sectional fluorescence microscopy images of the sample with and without induced heating, respectively. (**d**) Schematic illustration of body parts susceptible to the cold. (**e**) Infrared thermal camera images of the Cu–graphene on-skin joule heating element on the ear and hand of a human (scale bar: ~1cm).

Finally, we applied our on-skin electrodes for the heating element in on-demand drug delivery and frostbite prevention to demonstrate the multifunctional utility. The flexible electrodes based on light transdermal activation are promising candidates for an on-demand delivery, where Cu–graphene films can be used to convert light to heat. We placed the on-skin electrodes on pig skin and chose rhodamine B as the model drug. The film was heated by imposing light signals at a temperature of about 40 °C for 1 h (Figure 6a). Figure 6b,c show the cross-sectional fluorescent microscopic images of the drug delivered to the skin without and with the on-skin electrode. Compared with smooth skin, rhodamine B permeated more area in the skin with the on-skin electrodes, indicating its light-to-heat character can promote transdermal delivery of the drug. These initial findings serve as a proof of concept for the device’s potential in drug delivery. However, given the requirements for biomedical translation, a comprehensive biosafety evaluation is imperative. Our subsequent research will be dedicated to systematically evaluating the device for biomedical applications. In addition, we explored the potential applications of the proposed on-skin electrodes for helping people in cold environments (Figure 6d). Due to the strain-resilient ultrathin performance, we can conveniently attach the electrodes to the ear and hand that are vulnerable to cold. Figure 6e demonstrates that the devices can maintain stable thermal stimulations by the joule heating effect when humans typically walk. A key challenge in the field of flexible electronics is fabricating conformable conductive layers on various substrates. Our approach addresses this by utilizing tattoo paper, which readily forms stable interfaces with human skin and other materials, in conjunction with kirigami cutting techniques. The resulting bilayer electrode, featuring both graphene and metal layers, provides dual conduction pathways. This design not only improves electrical stability but also capitalizes on the photothermal effect of graphene, a feature absent in single-metal electrodes.

## 4. Conclusions

In summary, we reported a novel crack control strategy to fabricate strain-resilient thin-film conductors featuring simple preparation and available various substrates. The Cu–graphene layered design can improve the electromechanical functionality of the conductors, enabling strain-insensitive conductivity under stretching and bending. We used the proposed method to create on-skin electrodes that are water-resistant, interfacially tough, with good interfacial toughness, and highly conductive. Based on the triboelectrification and joule heating effects, an integrated electronic skin was developed to realize an intelligent dialing communication system and electrically active heaters. They can call a cell phone on human skin through the self-powered on-skin electrodes, promote transdermal delivery of the drug, and heat parts of the body susceptible to cold. These findings may attract extensive attention to flexible strain-resilient conductors and provide a design for meeting the challenges in electronic skin systems.

## Data Availability

The original contributions presented in this study are included in the article. Further inquiries can be directed to the corresponding author.

## Supplementary Materials

The following supporting information can be downloaded at: https://www.mdpi.com/article/10.3390/mi17030312/s1. Figure S1. Fabrication sequence of the Cu-graphene film conductor. Figure S2. The cross-sectional view of Cu-graphene film. Figure S3. Raman spectra of the honeycomb graphene. Figure S4. Measurements of adhesion forces for thin-film electrodes on different substrates. Figure S5. Electrical behavior of different films under different strain. Figure S6. Resistance of the graphene film. Figure S7. Illustration of conductive path. (a) Cu film. (b) Cu/graphene composite film. Figure S8. A uniaxial tensile test of the thin-film electrode. Figure S9. Schematic comparison of strategies to enhance the flexibility of conductive films. (a) A flat rigid conductive film. (b) Increasing the interfacial malleability by the tattoo film with folding structures. Figure S10. The strain–resistance curve of the conductive film. Figure S11. Stability test of the Cu-graphene film conductor under repeated bending strain. Figure S12. Photographs of Cu-graphene films on a finger connected by a red LED in response to bending and recovering deformation (scale bar: ~2 cm). Figure S13. Illustration of the kirigami model. Figure S14. The strain–resistance curve of the kirigami-patterned conductive film. Figure S15. Repeatability stability of different conductive films. Figure S16. Output voltage of the device when actuated with a pressure. Figure S17. Output voltage signals of the device under (a) non-contact and (b) contact conditions. Figure S18. The IR image of the Cu-graphene electrical heater responses at the applied voltage of 1.7 V. Figure S19. The current under different driving voltages (0.5 V–1.7 V). Figure S20. Evaluation of device wearing comfort and skin compatibility. Figure S21. A Large-area conductive thin films for thermal insulation applications.

## References

[1] Xu C., Solomon S.A., Gao W. (2023). Artificial Intelligence-Powered Electronic Skin. Nat. Mach. Intell..

[2] Zhao K., Zhao Y., Qian R., Ye C. (2023). Recent progress on tattoo-like electronics: From materials and structural designs to versatile applications. Chem. Eng. J..

[3] Niu W., Tian Q., Liu Z., Liu X. (2023). Solvent-Free and Skin-Like Supramolecular Ion-Conductive Elastomers with Versatile Processability for Multifunctional Ionic Tattoos and On-Skin Bioelectronics. Adv. Mater..

[4] Entifar S.A.N., Entifar N.A.E., Wibowo A.F., Kim J.H., br Sembiring Y.S., Saputro J.M.W., Kim H.-G., Kim J.-O., Xie G., Oh J. (2025). Extremely-low electrical-hysteresis hydrogels for multifunctional wearable sensors and osmotic power generators. Chem. Eng. J..

[5] Lee S., Kim T., Kim S., Park H., Lee J., Shim B.S. (2024). Electroactive Melanin Tattoo Inks for Effectively Reducing Skin Impedances. ACS Appl. Polym. Mater..

[6] Entifar N.A.E., Azizi M.J., Kim G.W., Entifar S.A.N., Slamet M.N., Kim J.H., br Sembiring Y.S., Wibowo A.F., Oh J., Lee J. (2025). High-performance PVA/xanthan gum hydrogel via dual cross-linking with ionic treatment for wearable sensing and hydrovoltaic energy generation. Chem. Eng. J..

[7] Li X., Zhang J., Shi B., Li Y., Wang Y., Shuai K., Li Y., Ming G., Song T., Pei W. (2025). Freestanding Transparent Organic–Inorganic Mesh E-Tattoo for Breathable Bioelectrical Membranes with Enhanced Capillary-Driven Adhesion. ACS Appl. Mater. Interfaces.

[8] Cai Y., Wang G., Mei Y., Zhao D., Peng J., Sun N., Zhang H., Han J., Yang Y. (2022). Self-healable, super-stretchable and shape-adaptive triboelectric nanogenerator based on double cross-linked PDMS for electronic skins. Nano Energy.

[9] Guo J., Liu X., Sun Z., Zheng X., Sung H.-K., Yao Z., Li Y., Li Y. (2024). An intelligent dual-sensing e-skin system for pressure and temperature detection using laser-induced graphene and polydimethylsiloxane. Mater. Des..

[10] Zhang Q., Ma L., Xue T., Tian J., Fan W., Liu T. (2023). Flame-retardant and thermal-protective polyimide-hydroxyapatite aerogel fiber-based composite textile for firefighting clothing. Compos. Part B Eng..

[11] Ying W.B., Yu Z., Kim D.H., Lee K.J., Hu H., Liu Y., Kong Z., Wang K., Shang J., Zhang R. (2020). Waterproof, highly tough, and fast self-healing polyurethane for durable electronic skin. ACS Appl. Mater. Interfaces.

[12] Ou Y., Lv J., Liu J., Chen S., Liu Y., Liu X. (2025). Surface Fluorination of Silicone Rubber with Enhanced Stain Resistance and Slip Properties. ACS Appl. Mater. Interfaces.

[13] Qu Y.X., Xia Q.Q., Li L.T., Cao C.F., Zhang G.D., Castignolles P., Bae J., Song P., Gao J.F., Tang L.C. (2024). Rational Design of Oil-Resistant and Electrically Conductive Fluorosilicone Rubber Foam Nanocomposites for Sensitive Detectability in Complex Solvent Environments. ACS Nano.

[14] Jung D., Kim Y., Lee H., Jung S., Park C., Hyeon T., Kim D.H. (2023). Metal-Like Stretchable Nanocomposite Using Locally-Bundled Nanowires for Skin-Mountable Devices. Adv. Mater..

[15] Chen G., Liang X., Men X., Liu L., Wang F., Bao X., Zhang H. (2023). Enhancing thermal conductivity and chemical protection of bacterial cellulose/silver nanowires thin-film for high flexible electronic skin. Int. J. Biol. Macromol..

[16] Haridas Cp A., Pillai S.K., Naskar S., Mondal T., Naskar K. (2024). Polyurethane/Carbon Nanotube-Based ThermoSense Electronic Skin: Perception to Decision Making Aided by Internet of Things Brain. ACS Appl. Mater. Interfaces.

[17] Yu X., Adronov A. (2025). Facile Preparation of Carbon Nanotube-Based Skin-Like Pressure Sensors. Small.

[18] Qi D., Zhang K., Tian G., Jiang B., Huang Y. (2021). Stretchable electronics based on PDMS substrates. Adv. Mater..

[19] Ha T., Tran J., Liu S., Jang H., Jeong H., Mitbander R., Huh H., Qiu Y., Duong J., Wang R.L. (2019). A chest-laminated ultrathin and stretchable E-tattoo for the measurement of electrocardiogram, seismocardiogram, and cardiac time intervals. Adv. Sci..

[20] Qi D., Liu Z., Yu M., Liu Y., Tang Y., Lv J., Li Y., Wei J., Liedberg B., Yu Z. (2015). Highly stretchable gold nanobelts with sinusoidal structures for recording electrocorticograms. Adv. Mater..

[21] Fan J.A., Yeo W.-H., Su Y., Hattori Y., Lee W., Jung S.-Y., Zhang Y., Liu Z., Cheng H., Falgout L. (2014). Fractal design concepts for stretchable electronics. Nat. Commun..

[22] Zhang Y., Xu S., Fu H., Lee J., Su J., Hwang K.-C., Rogers J.A., Huang Y. (2013). Buckling in serpentine microstructures and applications in elastomer-supported ultra-stretchable electronics with high areal coverage. Soft Matter.

[23] Liu Z., Wang X., Qi D., Xu C., Yu J., Liu Y., Jiang Y., Liedberg B., Chen X. (2016). High-Adhesion Stretchable Electrodes Based on Nanopile Interlocking. Adv. Mater..

[24] Ho M.D., Liu Y., Dong D., Zhao Y., Cheng W. (2018). Fractal gold nanoframework for highly stretchable transparent strain-insensitive conductors. Nano Lett..

[25] Kim J., Lee M., Shim H.J., Ghaffari R., Cho H.R., Son D., Jung Y.H., Soh M., Choi C., Jung S. (2014). Stretchable silicon nanoribbon electronics for skin prosthesis. Nat. Commun..

[26] Wang Y., Zhu C., Pfattner R., Yan H., Jin L., Chen S., Molina-Lopez F., Lissel F., Liu J., Rabiah N.I. (2017). A highly stretchable, transparent, and conductive polymer. Sci. Adv..

[27] Ershad F., Thukral A., Yue J., Comeaux P., Lu Y., Shim H., Sim K., Kim N.-I., Rao Z., Guevara R. (2020). Ultra-conformal drawn-on-skin electronics for multifunctional motion artifact-free sensing and point-of-care treatment. Nat. Commun..

[28] Huang Q., Zhu Y. (2021). Patterning of metal nanowire networks: Methods and applications. ACS Appl. Mater. Interfaces.

[29] Zhang R., Jiang J., Wu W. (2022). Scalably nanomanufactured atomically thin materials-based wearable health sensors. Small Struct..

[30] Choi S., Park J., Hyun W., Kim J., Kim J., Lee Y.B., Song C., Hwang H.J., Kim J.H., Hyeon T. (2015). Stretchable heater using ligand-exchanged silver nanowire nanocomposite for wearable articular thermotherapy. ACS Nano.

[31] Boley J.W., White E.L., Chiu G.T.C., Kramer R.K. (2014). Direct writing of gallium-indium alloy for stretchable electronics. Adv. Funct. Mater..

[32] Jeong S.H., Hagman A., Hjort K., Jobs M., Sundqvist J., Wu Z. (2012). Liquid alloy printing of microfluidic stretchable electronics. Lab A Chip.

[33] Li X., Zhu P., Zhang S., Wang X., Luo X., Leng Z., Zhou H., Pan Z., Mao Y. (2022). A self-supporting, conductor-exposing, stretchable, ultrathin, and recyclable kirigami-structured liquid metal paper for multifunctional E-skin. ACS Nano.

[34] Park J.-E., Kang H.S., Baek J., Park T.H., Oh S., Lee H., Koo M., Park C. (2019). Rewritable, printable conducting liquid metal hydrogel. ACS Nano.

[35] Lou Y., Liu H., Zhang J. (2020). Liquid metals in plastics for super-toughness and high-performance force sensors. Chem. Eng. J..

[36] Bartlett M.D., Fassler A., Kazem N., Markvicka E.J., Mandal P., Majidi C. (2016). Stretchable, high-k dielectric elastomers through liquid-metal inclusions. Adv. Mater..

[37] Cho C., Kang P., Taqieddin A., Jing Y., Yong K., Kim J.M., Haque M.F., Aluru N.R., Nam S. (2021). Strain-resilient electrical functionality in thin-film metal electrodes using two-dimensional interlayers. Nat. Electron..

[38] Hu H., Guo X., Zhang Y., Chen Z., Wang L., Gao Y., Wang Z., Zhang Y., Wang W., Rong M. (2023). Elasto-plastic design of ultrathin interlayer for enhancing strain tolerance of flexible electronics. ACS Nano.

[39] Zhuo F., Zhou J., Liu Y., Xie J., Chen H., Wang X., Luo J., Fu Y., Elmarakbi A., Duan H. (2023). Kirigami-inspired 3D-printable MXene organohydrogels for soft electronics. Adv. Funct. Mater..

[40] Gu J., Jung Y., Ahn J., Ahn J., Choi J., Kang B., Jeong Y., Ha J.-H., Kim T., Jung Y. (2024). Auxetic kirigami structure-based self-powered strain sensor with customizable performance using machine learning. Nano Energy.

[41] Liu Y., Zheng M., O’Connor B., Dong J., Zhu Y. (2022). Curvilinear soft electronics by micromolding of metal nanowires in capillaries. Sci. Adv..

[42] Yang T., Li X., Jiang X., Lin S., Lao J., Shi J., Zhen Z., Li Z., Zhu H. (2016). Structural engineering of gold thin films with channel cracks for ultrasensitive strain sensing. Mater. Horiz..

[43] Vohra A., Schlingman K., Carmichael R.S., Carmichael T.B. (2018). Membrane-interface-elastomer structures for stretchable electronics. Chem.

[44] Matsuhisa N., Jiang Y., Liu Z., Chen G., Wan C., Kim Y., Kang J., Tran H., Wu H.C., You I. (2019). High-transconductance stretchable transistors achieved by controlled gold microcrack morphology. Adv. Electron. Mater..

[45] Chen G., Matsuhisa N., Liu Z., Qi D., Cai P., Jiang Y., Wan C., Cui Y., Leow W.R., Liu Z. (2018). Plasticizing silk protein for on-skin stretchable electrodes. Adv. Mater..

[46] Xu F., Zhu Y. (2012). Highly conductive and stretchable silver nanowire conductors. Adv. Mater..

[47] Chun K.-Y., Oh Y., Rho J., Ahn J.-H., Kim Y.-J., Choi H.R., Baik S. (2010). Highly conductive, printable and stretchable composite films of carbon nanotubes and silver. Nat. Nanotechnol..

[48] Liu Y., Xu X., Wei Y., Chen Y., Gao M., Zhang Z., Si C., Li H., Ji X., Liang J. (2022). Tailoring silver nanowire nanocomposite interfaces to achieve superior stretchability, durability, and stability in transparent conductors. Nano Lett..

[49] Kang D., Pikhitsa P.V., Choi Y.W., Lee C., Shin S.S., Piao L., Park B., Suh K.-Y., Kim T.-I., Choi M. (2014). Ultrasensitive mechanical crack-based sensor inspired by the spider sensory system. Nature.

[50] Tao X., Chen X., Wang Z.L. (2023). Design and synthesis of triboelectric polymers for high performance triboelectric nanogenerators. Energy Environ. Sci..

[51] Cao X., Xiong Y., Sun J., Xie X., Sun Q., Wang Z.L. (2023). Multidiscipline applications of triboelectric nanogenerators for the intelligent era of internet of things. Nano-Micro Lett..

[52] Yang H., Li Q., Zhang X., Li X., Yang Q., Hu Y., Xi Y., Wang Z.L. (2022). High-sensitive and ultra-wide spectrum multifunctional triboelectric acoustic sensor for broad scenario applications. Nano Energy.

[53] Gogurla N., Kim Y., Cho S., Kim J., Kim S. (2021). Multifunctional and ultrathin electronic tattoo for on-skin diagnostic and therapeutic applications. Adv. Mater..

[54] Xu Y., Zhao G., Zhu L., Fei Q., Zhang Z., Chen Z., An F., Chen Y., Ling Y., Guo P. (2020). Pencil–paper on-skin electronics. Proc. Natl. Acad. Sci. USA.

[55] Mei C., Wang N., Zhu X., Wong K.H., Chen T. (2018). Photothermal-controlled nanotubes with surface charge flipping ability for precise synergistic therapy of triple-negative breast cancer. Adv. Funct. Mater..

