# Supplementary material for: A Stretchable Electronic Tattoo for Self-Powered Human–Machine Interfaces and Therapeutic Applications
Source: Micromachines (Basel). 2026 Feb 28;17(3):312. doi: 10.3390/mi17030312 (PMC13028016; doi:10.3390/mi17030312)
Supplement: Supplementary file 1 [file micromachines-17-00312-s001.zip › micromachines-4148679-supplementary.pdf]

Supplementary Materials

A Stretchable Electronic Tattoo for Self-Powered Human-Machine Interfaces and Therapeutic Applications

Rumeng Shao [1], Yixuan Zhang [1], Ya Chang [1], Chuanbo Li [1,2] and Yang Wang [1,2,*]

1 School of Science, Minzu University of China, Beijing 100081, China
2 Optoelectronics Research Centre, Minzu University of China, Beijing 100081, China
* Correspondence: yangwang@muc.edu.cn

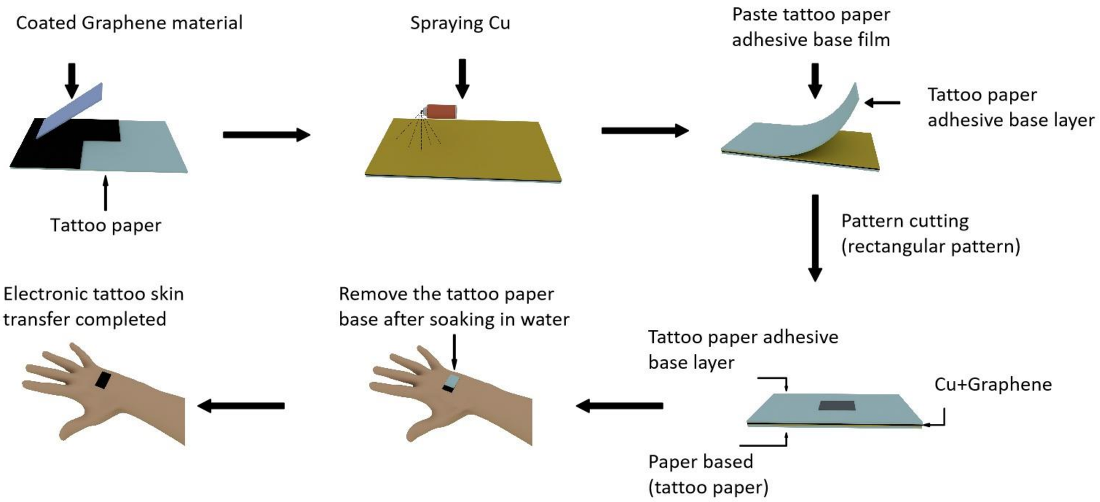

Figure S1. Fabrication sequence of the Cu-graphene film conductor.

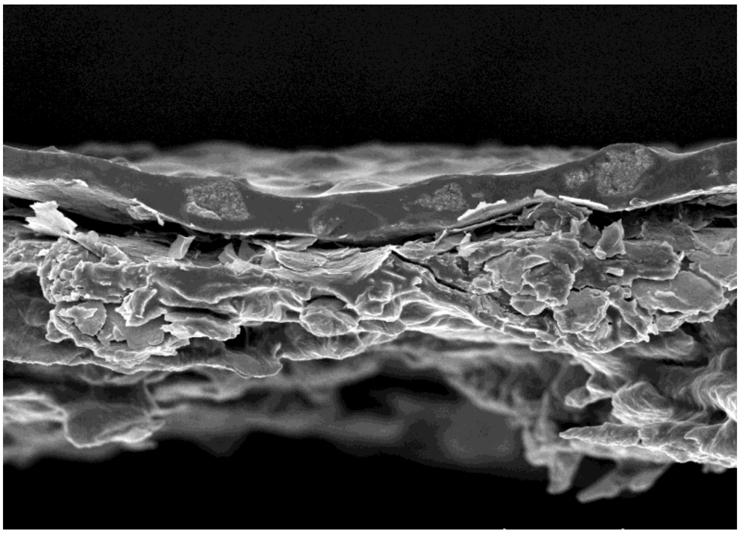

Figure S2. The cross-sectional view of Cu-graphene film.

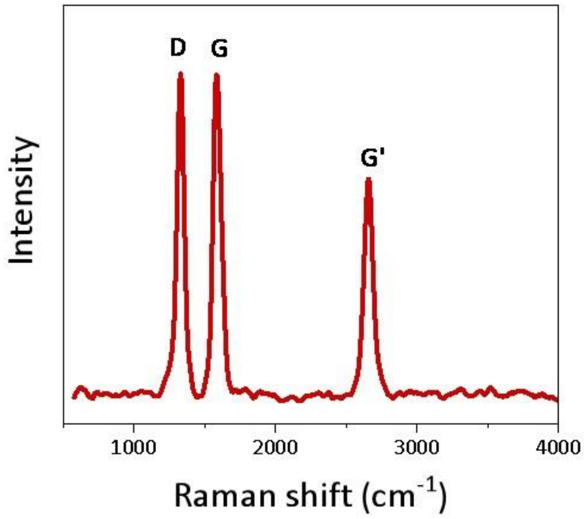

Figure S3. Raman spectra of the honeycomb graphene.

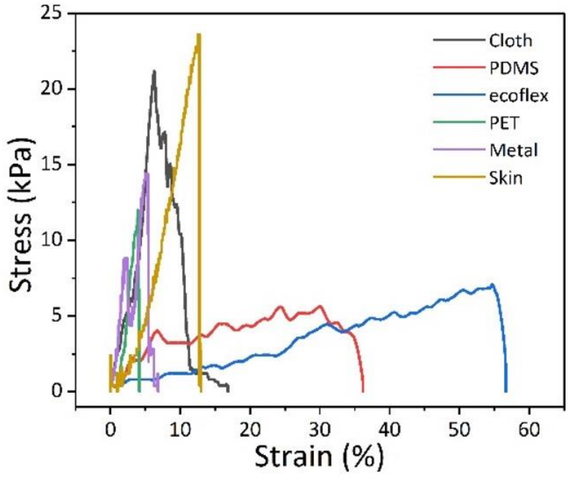

Figure S4. Measurements of adhesion forces for thin-film electrodes on different substrates.

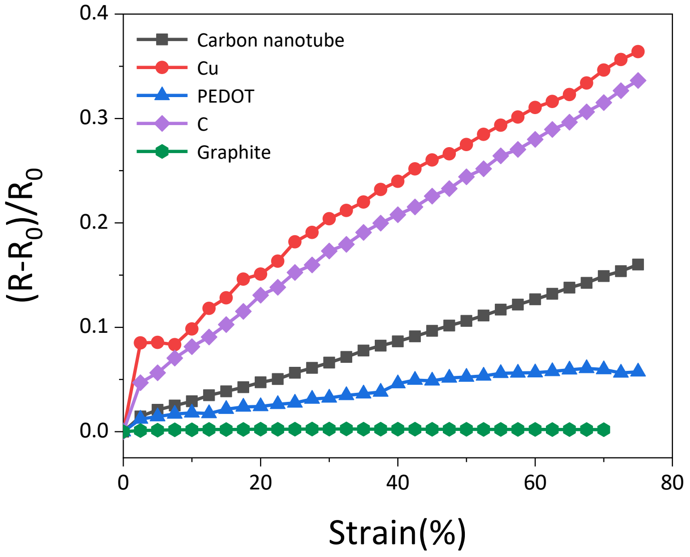

Figure S5. Electrical behavior of different films under different strain.

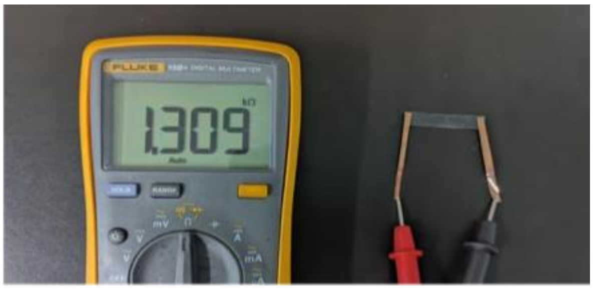

Figure S6. Resistance of the graphene film.

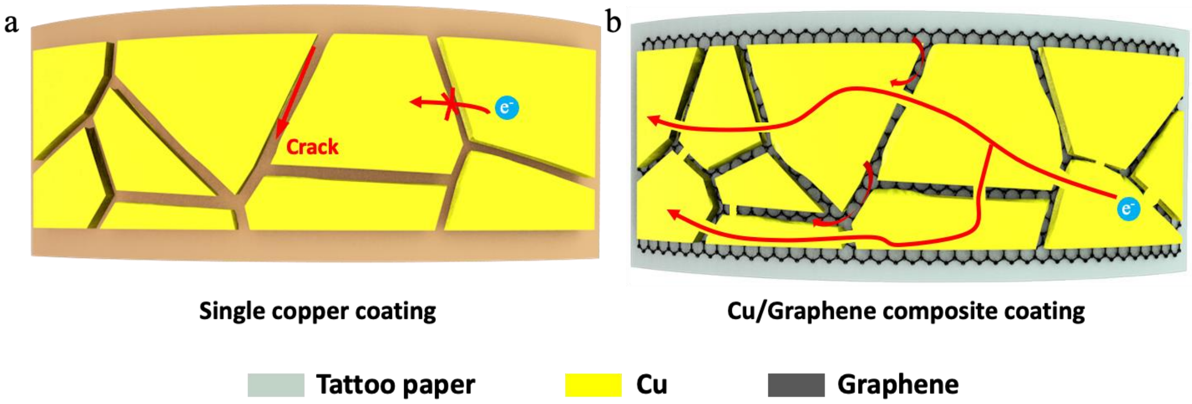

Figure S7. Illustration of conductive path. (a) Cu film. (b)Cu/graphene composite film.

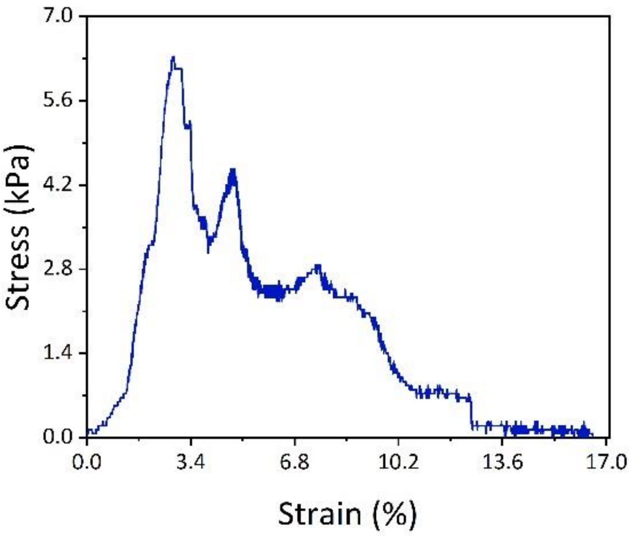

Figure S8. A uniaxial tensile test of the thin-film electrode.

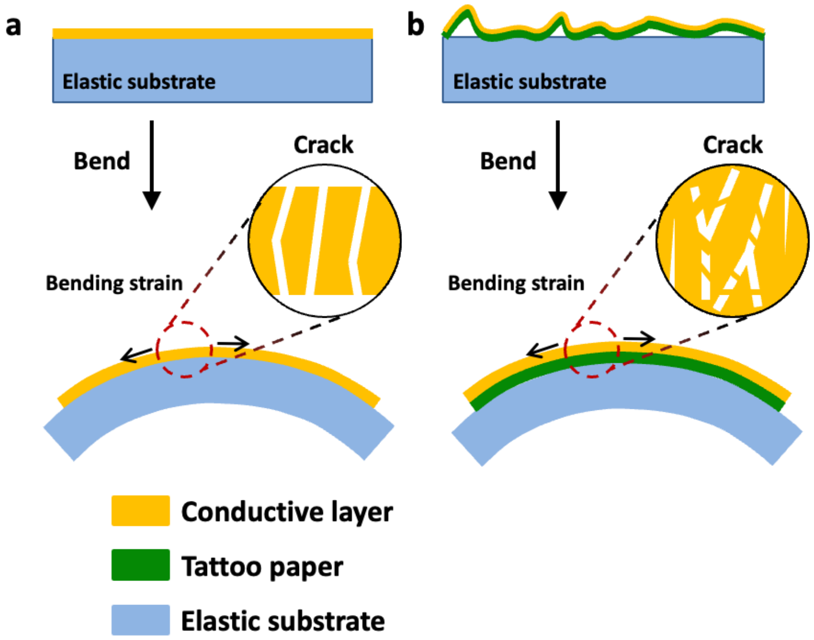

Figure S9. Schematic comparison of strategies to enhance the flexibility of conductive films. (a) A flat rigid conductive film. (b) Increasing the interfacial malleability by the tattoo film with folding structures.

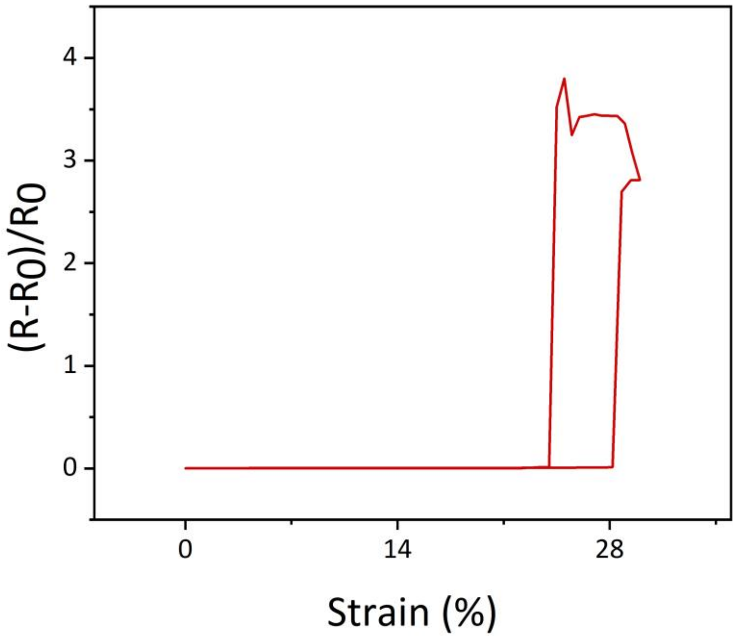

Figure S10. The strain–resistance curve of the conductive film.

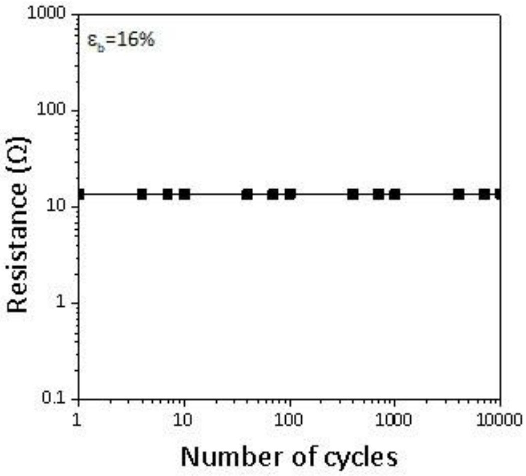

Figure S11. Stability test of the Cu-graphene film conductor under repeated bending strain.

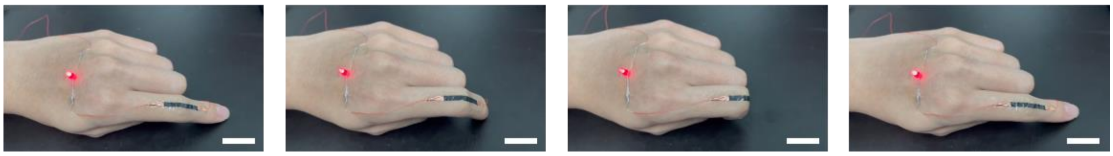

Figure S12. Photographs of Cu-graphene films on a finger connected by a red LED in response to bending and recovering deformation (scale bar: ~2 cm).

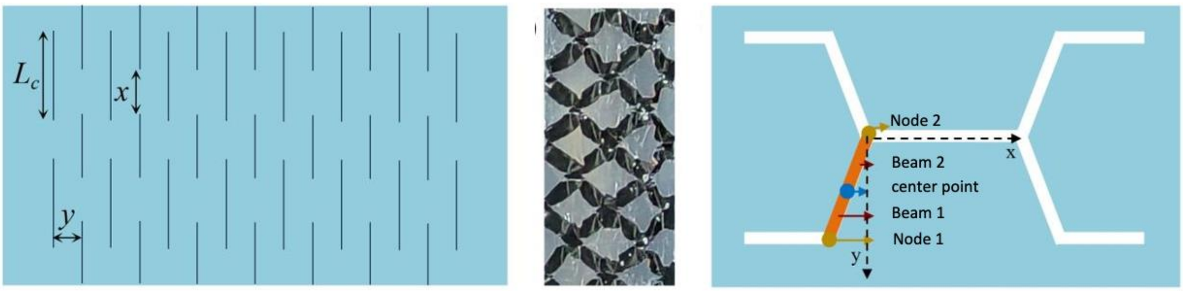

Figure S13. Illustration of the kirigami model.

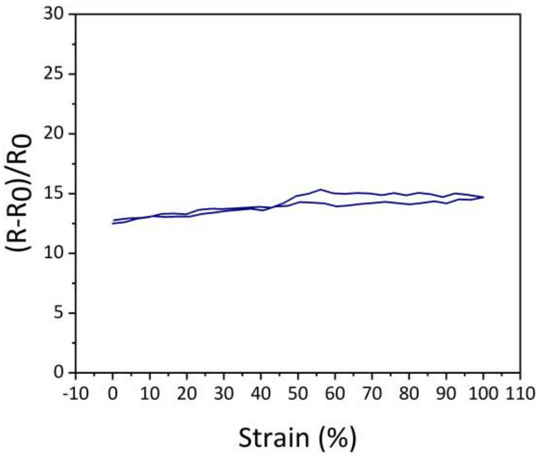

Figure S14. The strain–resistance curve of the kirigami-patterned conductive film.

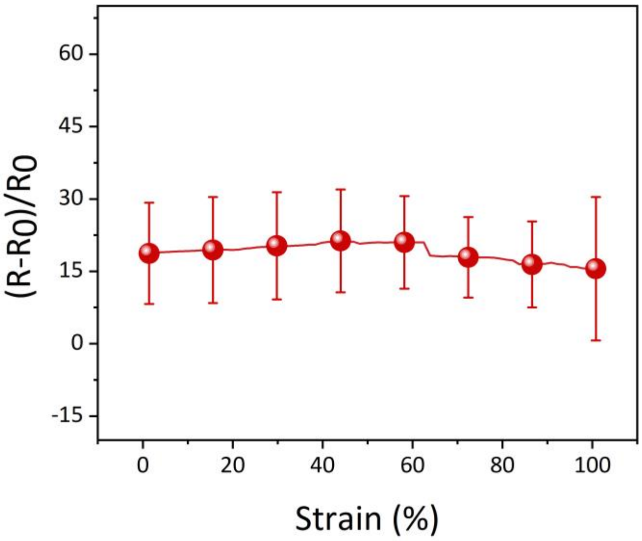

Figure S15. Repeatability stability of different conductive films.

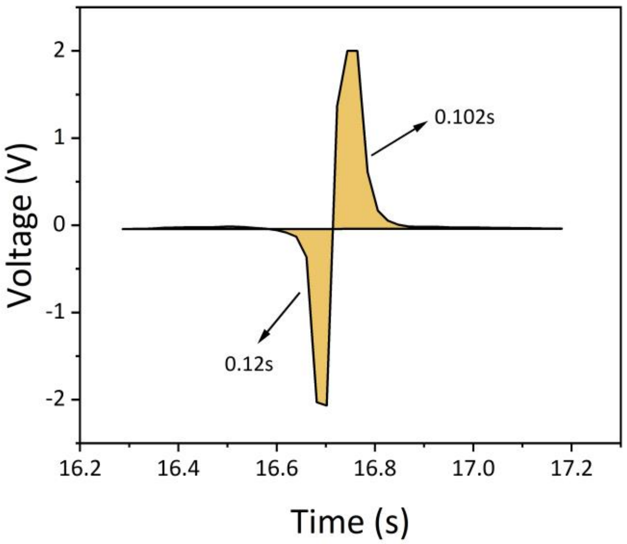

Figure S16. Output voltage of the device when actuated with a pressure.

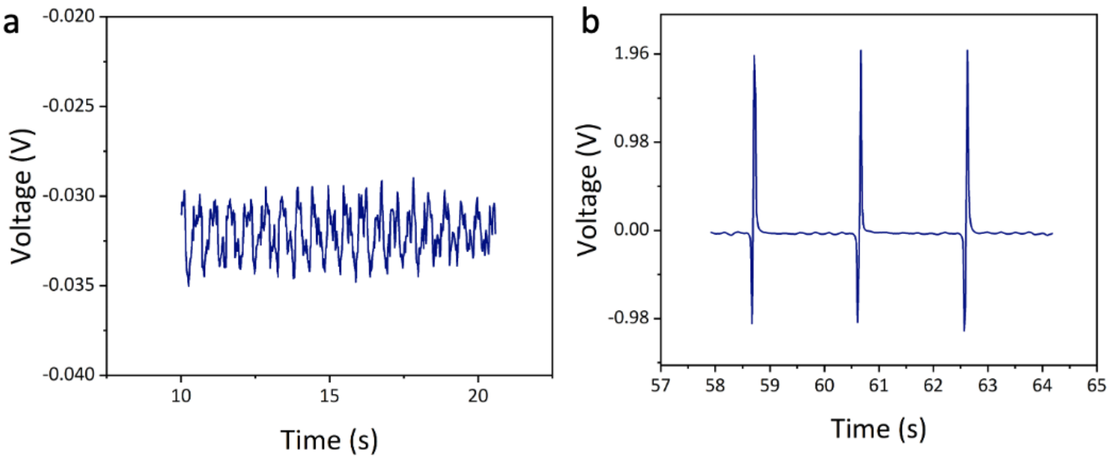

Figure S17. Output voltage signals of the device under (a) non-contact and (b) contact conditions.

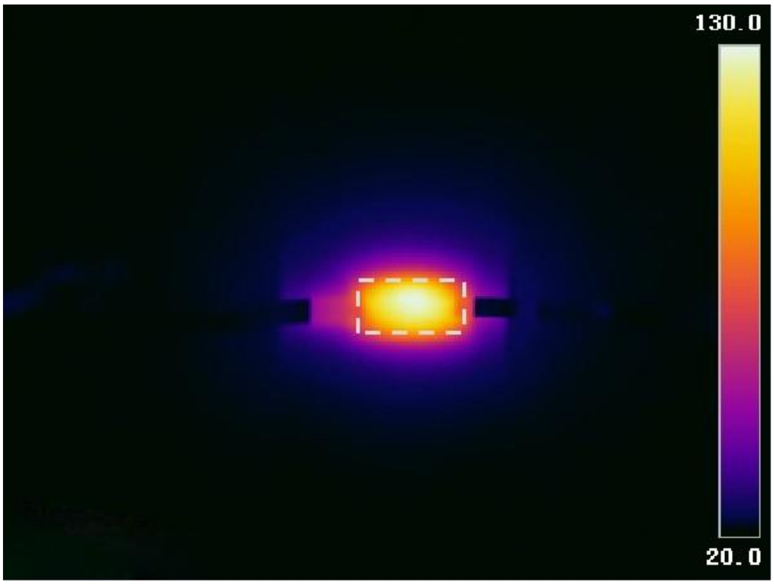

Figure S18. The IR image of the Cu-graphene electrical heater responses at the applied voltage of 1.7 V.

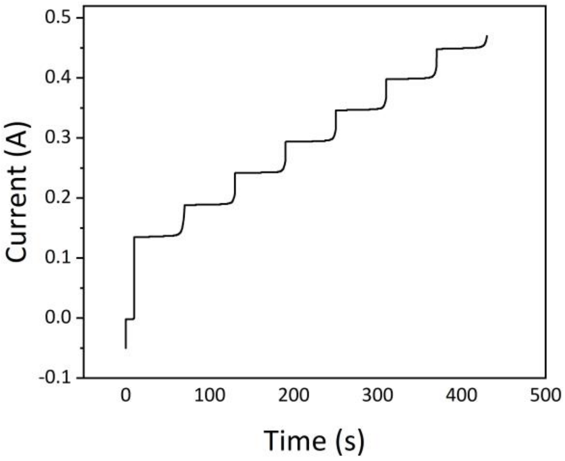

Figure S19. The current under different driving voltages (0.5 V–1.7 V).

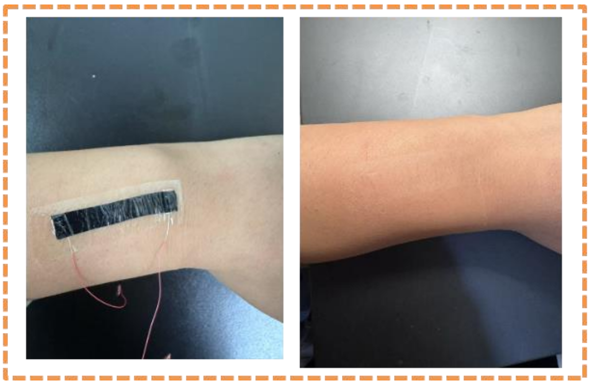

Figure S20. Evaluation of device wearing comfort and skin compatibility.

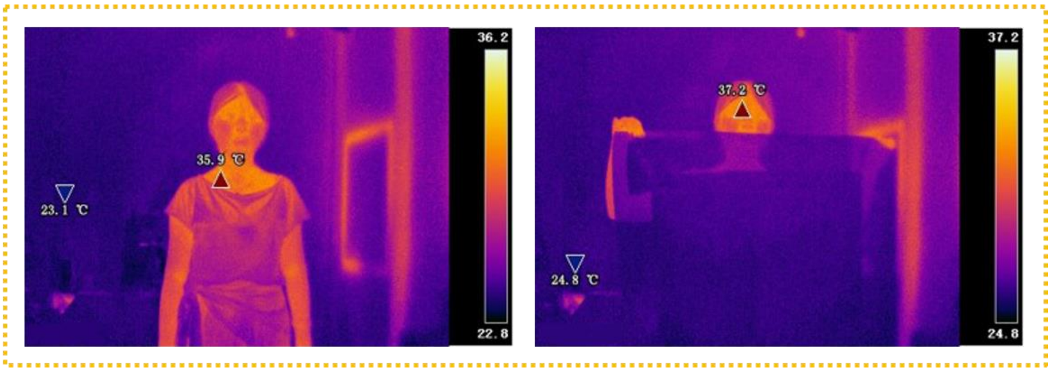

Figure S21. A Large-area conductive thin films for thermal insulation applications.
